# Prevalence of surgical site infection and its associated factors after cesarean section in Ethiopia: systematic review and meta-analysis

**DOI:** 10.1186/s12884-020-03005-8

**Published:** 2020-05-20

**Authors:** Temesgen Getaneh, Ayenew Negesse, Getenet Dessie

**Affiliations:** 1 Department of Midwifery, College of Health Science, Debre Markos University, P.O. Box 269, Debre Markos, Ethiopia; 2 Department of Human Nutrition and Food Sciences, College of Health Science, Debre Markos University, Debre Markos, Ethiopia; 3 Center of excellence in Human Nutrition, School of Human Nutrition, Food Science and Technology, Hawassa University, Hawassa, Ethiopia; 4 Department of Nursing, School of Health science, College of Medicine and Health Science, Bahr Dar University, Bahir Dar, Ethiopia

**Keywords:** Cesarean section, Prevalence, Surgical site infection, Ethiopia

## Abstract

**Background:**

Surgical site infection (SSI) affects nearly one third of patients who have undergone a surgical procedure. It is a significant and substantial cause of surgical patient morbidity and mortality later with human and financial costs threat. There are fragmented and pocket studies which reported the prevalence of SSI among mothers who experienced for cesarean section and its risk factors. However, there is no any solid evidence established at the national level; which was also the interest of the authors to fill this gap. Therefore, this systematic review and meta-analysis aimed to estimate the pooled prevalence of SSI after cesarean section and its associated factors at national level.

**Methods:**

Original articles were searched in PubMed, MEDLINE, EMBASE, CINAHL, Google Scholar, HINARI portal, and Cochrane Library. All observational studies defined outcome of variable “SSI as infection related to an operation procedure that occur at or near surgical incision within 30 days of operation or after one year if an implant is placed” were considered. Data were extracted using standard data extraction excel spread sheet checklists developed according to 2014 Joanna Briggs Institute Reviewers’ Manual after the quality was assessed through Newcastle–Ottawa quality assessment scale. The *I*^*2*^ statistic was used to quantify heterogeneity across studies. Funnel plot asymmetry and Egger’s tests were used to check for publication bias. A fixed effect model was used to estimate the pooled prevalence of SSI. Odds Ratio (OR) with 95% Confidence Interval (CI) was also considered to determine the association of identified variables with SSI. Statistical analysis was conducted using STATA version 14 software.

**Result:**

From initial 179 identified articles, 11 were eligible for inclusion in the final systematic review and meta-analysis. Studies with a score of 6 and above were included for final analysis. All included studies were institutional based cross sectional. The pooled estimate of SSI after cesarean section in Ethiopia was 9.72% (95%CI: 8.38, 11.05). Premature rapture of membrane (PROM) > 12 h (OR = 5.32, 95%CI: 3.61, 7.83), duration of labor> 24 h (OR = 3.67, 95%CI: 2.45, 5.48), chorioamnionitis (OR = 9.11, 95%CI: 5.21, 15.93), anemia (OR = 4.56, 95%CI: 2.88, 7.22) and having vertical skin incision (OR = 4.17, 95%CI: 2.90, 6.02) had increased odds of developing SSI after cesarean section.

**Conclusion:**

The prevalence of SSI after cesarean section in Ethiopia was high compared with the sphere standards of communicable disease control (CDC) guidelines for SSI after cesarean section. Therefore, Ministry of Health with its stake holders should give special emphasis on community and institution based programs in manner to prevent prolonged labor, PROM, chorioamnionitis and anemia which will also have synergistic impact on SSI after cesarean section. Moreover, there is also a call to health professionals not to use vertical incision as primary option of cesarean section to reduce the risk of developing surgical site infection among mothers.

## Background

Health care-associated infections (HAI) are acquired by patients while receiving care, and represent the most frequent adverse event affecting patient safety worldwide [1]. It is estimated that hundreds of millions of patients are affected by HAIs each year, leading to significant mortality and financial losses for health systems [2, 3]. Currently evidences shows that SSI is the most surveyed and frequent type of HAI in low and middle-income countries and affects nearly one third of patients who have undergone a surgical procedure [4]. SSI is infection related to an operation procedure that occurs at or near surgical incision within 30 days of operation or after 1 year if an implant is placed. It can be either incision (superficial: involving the skin and subcutaneous tissues and deep: involving the deeper soft tissues of the incision, such as muscle or fascia) or organ space (involving any part of the anatomy other than the incised body layers (skin, fascia, and muscle layers)) [5].

The number of surgical procedures performed now a day globally continues to rise, and surgical patients are initially seen with increasingly complex comorbidities. Cesarean section is one of the most commonly practiced surgical procedure [6]. Potential complications such as: SSI that associated with any type of surgical procedure including cesarean section represents a well-known significant cause of surgical patient morbidity and mortality with consecutive human and economic losses. Despite of SSI is the most preventable HAIs using evidence-based strategies, still the problem is represented with high prevalence (11.7%) which accounts for high human and financial costs [7]. A narrative review conducted in Sub-Sharan Africa (SSA) reported SSI after cesarean section was timely increasing (15.6%) [8]. SSI patients required prolonged hospitalization, reoperation and readmission [9]. The majority of infection-associated costs arise from prolonged hospitalization, with additional expenditure attributable to medical staff and treatment. As the demand for surgical procedures rises, the incidence and associated costs of SSI will likely escalate [10].

Many factors influence surgical wound healing and determine the presence of infection. Patient-related (endogenous) and process/procedural-related (exogenous) variables are the primary factors that increase the chance of SSI. There are also non modifiable variables such as age and gender that contribute for high prevalence of SSI. On the other hand there are also other potential factors, such as good nutritional status, avoiding tobacco use, correct use of antibiotics and the intraoperative technique that can increase the likelihood of positive surgical outcome [7].

The rate of cesarean section in Ethiopia was vary according to different individual studies report with a range from 11 to 34.4% [11, 12], but based on the Ethiopian Demographic Health Survey of 2016, it was 2% [13]. In Ethiopia, there are pocket and fragmented studies across regions that explore about the prevalence of SSI after cesarean section and associated factors among mothers [14–24]. However the studies were inconclusive and there was no any concrete scientific evidence established at national level. Therefore, this systematic review and meta-analysis aimed to estimate the pooled prevalence of SSI after cesarean section and its associated factors (specifically labor, its comorbidity and type of incision related that can be modifiable as majority of included articles searched) at the national level.

## Methods

### Searching strategies

This systematic review and meta-analysis was conducted in accordance with the Preferred Reporting Items for Systematic Reviews and Meta-Analyses Protocols (PRISMA-P) checklist guidelines (see Additional file 1) [25]. Potentially relevant articles were comprehensively searched using PubMed/MEDLINE, EMBASE, CINAHL, Google Scholar, HINARI portal (which includes the SCOPUS, African Index Medicus, and African Journals Online databases), and Cochrane Library. In addition, related articles found from review of the grey literature available on local shelves, institutional repositories and from reviewing the cross-reference list of already identified articles were also systematically reviewed. Electronic database searches were conducted from 5 May to 20 June 2019. The key terms used for the PubMed database searches were: “Prevalence”, “magnitude”, “surgical site”, “infection”, “cesarean section”, “associated factors” and “Ethiopia”. Endnote citation manager software version X7 for Windows was utilized to collect and organize search outcomes and for removal of duplicate articles.

#### Population

All the reproductive age women (15–49 years) who gave birth at least once through cesarean section were the population.

#### Exposure (E)

Mothers who had cesarean section and exposed for the risk of developing SSI. **Outcome (O):** SSI as infection related to an operation procedure that occurs at or near surgical incision within 30 days of operation or after 1 year if an implant placed was the outcome of interest.

### Eligibility criteria

#### Inclusion criteria

This systematic review and meta-analysis considered all the studies which were conducted in all regional sates of Ethiopia that reported SSI after cesarean section and its associated factors. This systematic review and meta-analysis was not restricted based on publication conditions, publication time and study designs.

#### Exclusion criteria

Based on the eligibility criteria, we read their titles and abstracts. If studies were relevant for our review, we examined the full texts. Those papers which did not fully accessed at the time of our search process were excluded after contact was attempted with the principal investigator through email at least two times. The reason for the exclusion of these articles is that we are unable to assess the quality of each article in the absence of their full texts. Moreover, studies which did not report our outcome of interest were excluded after reviewing their full texts. Studies which did not report SSI and its associated factors were also excluded from the final meta-analysis. Once more, studies with poor quality as per settled criteria of reviewing the articles were also excluded from this systematic review and meta-analysis.

### Quality of assessment

The database search results were exported and duplicate articles were removed using EndNote software (version X7; Thomson Reuters, New York, NY). We used the Newcastle–Ottawa quality assessment scale adapted for cross-sectional studies [26, 27]. The scale is used to score the articles under three categories:

- Selection (score 0–5);
- Comparability (score 0–2); and
- Outcome (score 0–3); total score range 0–10.

The first section scored focuses on the methodological quality of each study which includes representativeness of the sample, the sample size, non-respondents and ascertainment of the exposure. The second section of the tool considers the comparability of different outcome groups in the study based on the study design and analysis in which confounding factors are controlled (i.e. the study controls for the most important factor and the study control for any additional factor). The last section of the tool is deal about the assessment of the outcome (include independent blind assessment, record linkage and self-report) and statistical analysis of the original study (i.e. the statistical test used to analyze the data is clearly described and appropriate, and measure of association is presented, including CI and *p*-value). Two authors assessed articles and scored for each primary studies. The third author was considered in case of disagreement. Then studies with a score of ≥6 out of 10 were considered for final analysis (taken as high quality after reviewing different relevant literatures). Identified articles with methodological problems and incomplete reporting of results in the full text were excluded from the final meta-analysis.

We assessed and evaluated the methodological quality and risk of bias in the studies that were selected using the 10-item rating scale developed Hoy et al. for prevalence studies (see Additional file 2) [28]. Sampling, data collection, reliability and validity of study tools, case definition, and prevalence periods were included in the tool. The rating scale categorized as having low risk of bias (“yes” answers to domain questions) or high risk of bias (“no” answers to domain questions) for each articles. Each study was assigned a score of 1 (Yes) or 0 (No) for each domain, and these scores were summed to provide an overall study quality score. Scores of 8–10 were considered as having a “low risk of bias”, 6–7 a “moderate risk”, and 0–5 a “high risk”. For the final risk of bias classification, disagreements between the reviewers were resolved via consensus.

### Data extraction procedure

Data were independently extracted by two authors using standardized data extraction format that developed according to 2014 Joanna Briggs Institute Reviewers’ Manual [29] .The tool includes Authors, Region, study setting, study year, study design, sampling technique, sample size and prevalence of SSI after cesarean section as well as factors associated with SSI after cesarean section. Articles that fulfilled the predefined criteria were used as a source of data for the final analysis.

### Outcome measurements

The first outcome of this systematic review and meta-analysis was the pooled prevalence of SSI after cesarean section. SSI is infection related to cesarean section that occur at or near surgical incision within 30 days of operation or after 1 year if an implant is placed [5]. For analysis of the second outcome (risk factors of SSI after cesarean section), we extracted data on factors that had been found to be related to SSI after cesarean section in the literature, such as the presence of PROM (defined as premature rapture of membrane after 28 weeks of complete gestational age and 1 h before the onset of true labor) (> 12 h), chorioamnionitis (defined as the infection of the fetal membranes; the chorion and amnion), duration of labor (the total duration of labor after initiation of true labor till delivery) (> 24 h), anemia (considered when preoperative hematocrit less than 30% or clinically diagnosed) and type of incision of the skin (vertical v_s_ transverse). Those articles considered for each variables used similar definition of terms and cut-off point.

### Data analysis

All the needed information on the articles were extracted using Microsoft Excel spreadsheet form and exported to STATA (version 14; Stata Corp, College Station, TX) for further analysis. The existence of statistical heterogeneity was assessed using Cochran’s Q statistic and quantifies using inverse variance (I^2^) and its corresponding *p* –value with fixed effect model of analysis (because the overall heterogeneity test was insignificant so it is better to use fixed effect model rather than random effect model). A value of 25, 50, and 75% were used to declare the heterogeneity test as low, medium and high heterogeneity [30]. Comparison of original articles using tabulation was computed to assess some clinical or methodological heterogeneity among the studies (Table-1). Visual examination of funnel plot asymmetry, Begg-Mazumdar Rank correlation tests and Egger’s regression tests were utilized to check for publication bias [31]. The estimated pooled prevalence of SSI after cesarean section was computed using forest plots with the 95% CI. Odds ratio was used to identify factors associated with SSI after cesarean section.

## Result

### Article selection

**Fig. 1:**
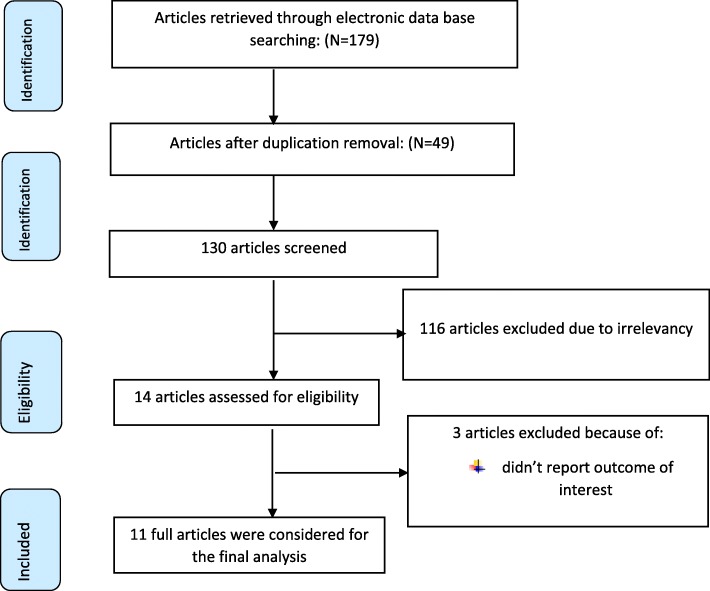
PRISMA flow diagram of included studies to estimate the pooled prevalence of SSI and its associated factors after cesarean section in Ethiopia 2009–2018

First of all, 179 articles related to SSI after cesarean section and associated factors were identified via electronic data bases. After reviewing their titles, 49 articles were removed due to duplication. The remaining 14 articles’ title and abstract were reviewed and, 116 articles were excluded with a reason of irrelevancy. From the remaining 14 articles, 3 articles [32–34] were excluded due to the absence of the outcome of interest. Finally 11articles were eligible and included for the final analysis (Fig. 1).

### Characteristics of the primary articles

In this meta-analysis, 4351 mothers were included with 100% response rate. All studies were published from 2011 to 2019. All articles were conducted through cross sectional study design. The smallest and the largest sample size was 131 [16] and 770 [22] respectively. Among the included studies, the lowest prevalence was reported from SNNPs [18] and the highest prevalence was reported from the Amhara regional state [22]. From the total studies, most of them were conducted at SNNPs [15, 18, 23] and Amhara regional states [14, 17, 21, 22]. All the included studies were conducted at Hospitals. According to Newcastle–Ottawa quality assessment scale adapted for cross-sectional studies quality scored was done for each articles fulfilled the inclusion criteria before actual data extraction was employed. As we stated above studies scored below 6 out of 10 were excluded from this systematic review and meta-analysis. Among 11 studies contributed for this review four of them scored 6 [15, 21, 22, 24], four of them scored 7 [14, 17, 19, 20], two of them 8 [18, 23] and single study scored 9 [16] after authors evaluated each articles according to criteria’s stated above on methodology part (Table 1).

**Table 1:** Descriptive summary of eleven studies included in the meta-analysis of SSI after cesarean section and associated factors in Ethiopia 2009–2018

Author	Region	study year	Sample size	Prevalence (%)	Factors listed	NOSS score
Mamo et al. [20]	Oromo	2015	384	9.4	PROM, prolonged labor, chorioamnionitis and vertical skin incision	7
Gelaw et al. [19]	AA	2017	474	8.4	PROM, anemia, chorioamnionitis and vertical skin incision	7
Aklilu et al. [15]	SNNPR	2017	325	12.9	Anemia	6
Amenu et al. [16]	Oromo	2009	770	11.4		9
Wedajo et al. [23]	SNNPR	2012	592	11		8
Azeze et al. [14]	Amhara	2018	383	7.8	Vertical skin incision	7
Gelaw et al. [18]	SNNPR	2016	384	6.8	Prolonged labor, anemia, and vertical skin incision	8
Mulu et al. [22]	Amhara	2011	131	13.7		6
Gedefaw et al. [17]	Amhara	2018	447	9.4	PROM, prolonged labor, chorioamnionitis and vertical skin incision	7
Rose et al. [21]	Amhara	2016	247	8.6	PROM	6
Wondemagegn et al. [24]	Tigray	2016	206	11.7	PROM, prolonged labor, chorioamnionitis and anemia	6

In regarding to risk bias assessment, seven studies (63.6%) had high quality scores while four studies (36.4%) had low quality scores (Table 2).

**Table 2:** Criteria used for scoring of risk bias assessment tool of included articles for the estimation of pooled prevalence of SSI after cesarean section and its associated factors in Ethiopia 2009–2018

Study ID	Representation	Sampling	Random selection	Non response bias	Data collection	Case Definition	Reliability and validity of study tool	Method of data collection	Prevalence period	Numerator and denominator	Summary Assessment
Mamo et al [20]	Low risk	Low risk	Low risk	Low risk	Low risk	High risk	Low risk	Low risk	Low risk	Low risk	Low risk
Gelaw et al [19]	High risk	Low risk	Low risk	Low risk	Low risk	Low risk	Low risk	Low risk	Low risk	Low risk	Low risk
Aklilu et al [15]	High risk	Low risk	High risk	Low risk	Low risk	High risk	High risk	High risk	low risk	Low risk	High risk
Amenu et al [23]	High risk	Low risk	High risk	Low risk	Low risk	High risk	High risk	High risk	low risk	Low risk	High risk
Wedajo et al [14]	Low risk	Low risk	Low risk	Low risk	Low risk	Low risk	Low risk	Low risk	Low risk	Low risk	Low risk
Azeze et al	Low risk	Low risk	Low risk	Low risk	Low risk	Low risk	Low risk	Low risk	Low risk	Low risk	Low risk
Gelaw et al [18]	Low risk	Low risk	Low risk	Low risk	Low risk	Low risk	Low risk	Low risk	Low risk	Low risk	Low risk
Mulu et al [22]	Low risk	Low risk	High risk	Low risk	High risk	High risk	High risk	Low risk	Low risk	Low risk	High risk
Gedefaw et al [17]	Low risk	Low risk	Low risk	Low risk	Low risk	Low risk	Low risk	Low risk	Low risk	Low risk	Low risk
Rose et al [21]	Low risk	Low risk	Low risk	Low risk	Low risk	Low risk	Low risk	Low risk	Low risk	Low risk	Low risk
Wondemagegn et al [24]	Low risk	Low risk	High risk	Low risk	High risk	High risk	High risk	Low risk	Low risk	Low risk	High risk

### Prevalence of SSI after cesarean section in Ethiopia

**Fig. 2:**
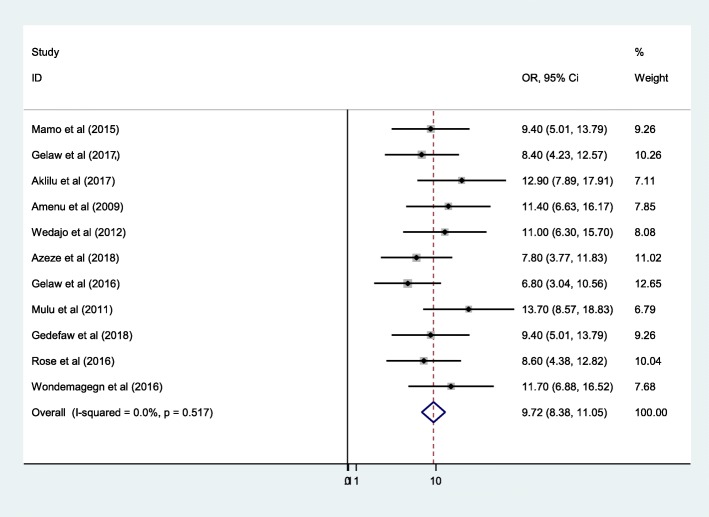
Forest plot of the pooled prevalence of SSI after cesarean section in Ethiopia 2009–2018

The pooled prevalence of SSI after cesarean section in Ethiopia was 9.72% (95%CI: 8.38, 11.05) (Fig. 2). In this systematic review and meta-analysis, the heterogeneity test showed that there was no any type of heterogeneity detected, I^2^ = 0.0%, *p*-value =0.517. The publication bias was also insignificant as suggested by objective test of Begg’s and Egger’s test, which showed with *p*-value of *p* = 0.392 and *p* = 0.293 respectively.

### Publication bias

**Fig. 3:**
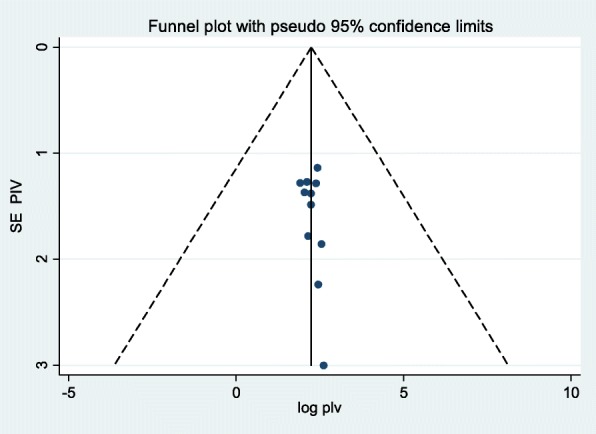
Meta funnel presentation of the prevalence of SSI after cesarean section, **2009–2018,** Ethiopia

In this systematic review and meta-analysis the symmetry of funnel plot and non-significance of Egger’s test indicated that publication bias was not significant. As demonstrated below in the funnel plot it shows symmetric (Fig. 3) and Egger’s test *p*-value =0.259 (95% CI: − 0.541- 1.907).

### Factors associated with SSI after cesarean section

As the studies included under this systematic review and meta-analysis were cross sectional study, all used OR as measure of association and the definition of variables. The association between PROM more than 12 h and SSI after cesarean section was reported in five articles [17, 19–21, 24]. All articles reported on effect PROM defined it as premature rapture of membrane 1 h before the onset of labor. Mothers with PROM more than 12 h were 5.32 times more likely to have SSI after cesarean section as compared with mothers with PROM ≤12 h, OR = 5.32 and (95%CI: 3.61, 7.83). The relation duration of labor more than 24 h and the risk of SSI after cesarean section were also reported in four included studies [17, 18, 20, 24].

**Fig. 4:**
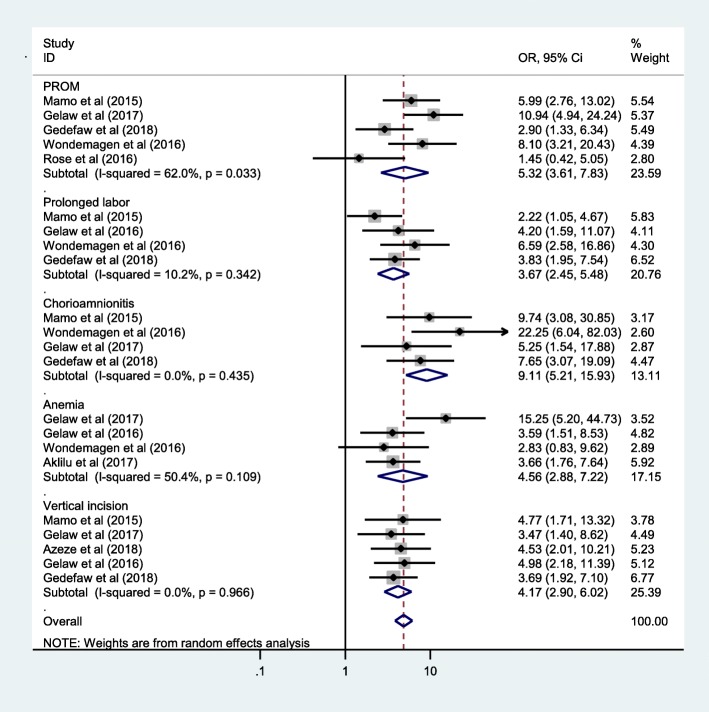
Forest plots which describe factors associated with SSI after cesarean section in Ethiopia 2009–2018

The likelihood of SSI after cesarean section was 3.67 times higher among mothers with duration of labor more than 24 h as compared with mothers whose labor duration was ≤24 h, OR = 3.67 and (95%CI: 2.45, 5.48). There was also significant relationship between SSI after cesarean section and chorioamnionitis as reported from four articles [17, 19, 20, 24]. Mothers with chorioamnionitis were 9.11 times more likely to develop SSI after cesarean section as compared with mothers without chorioamnionitis, OR = 9.11 and (95%CI: 5.21, 15.93). Anemia was also another contribution factor for SSI after caesarian section and their connection were reported in four articles [15, 18, 19, 24]. The risk of developing SSI after cesarean section were 4.56 times higher among anemic mothers as compared to non-anemic mothers,OR = 4.56 and (95%CI: 2.88, 7.22). Once more, vertical incision of skin was also another risk for SSI after cesarean section as their correlation reported by five articles [14, 17–20]. Mothers who had history of vertical skin incision during cesarean section had 4.17 times increased risk of SSI as compared with mothers who had history of transverse skin incision for cesarean section, OR = 4.17 and (95%CI: 2.90, 6.02) (Fig. 4).

## Discussion

This Systematic review and meta-analysis was conducted to identify the pooled prevalence of SSI after cesarean section and associated factors in Ethiopia. We found high prevalence of SSI after cesarean section in Ethiopia with an overall prevalence of 9.72% (95%CI: 8.38, 11.05). This systematic review and meta-analysis reported a higher prevalence as it was compared with the sphere standard of CDC guidelines of SSI (which was 5%) [35].

SSI after cesarean section is considered as an indication of quality of health care service. However, it represented with high figure that make the quality of health care service to be questionable in Ethiopia. Although several endogenous risk factors are there, it can be possibly explained by that limited and ineffective implementation of evidence based SSI prevention strategies recommended by CDC may increase the problems.

This includes limited and ineffective administering of antimicrobials before 1 h of procedure, prolonged preoperative admission of patients, longer duration of procedures and inability to prevent obstetric complications (PROM, chorioamnionitis) [36]. It was also much higher than among studies conducted in Nova Scotia and New Zealand reports of SSI prevalence; 2.7 and 5.2% [37, 38]respectively.

This systematic review and meta-analysis was lower as compared with a reports from India (8.9%,), England (9%), Norway (9.1%) and Nigeria (9.6%) [39–42]. It was also much lower than from reports of Jordan (14.4%), Malaysia (18.8%), a systematic review from Sub-Saharan Africa (15.6%) and Egypt (16.7%) [8, 43–45]. Hence, application of evidence based strategies should be there, like timely administration of appropriately selected prophylactic antibiotics, use of a chlorhexidine-alcohol based preparation, use of suture for skin closure, maintenance of glycemic control in the postoperative period, showering (full body) with soap (antimicrobial or non-antimicrobial) or an antiseptic agent on at least the night before the operative day, normothermia should be maintained in all patients, increased fraction of inspired oxygen should be administered during surgery and after extubation in the immediate postoperative period for patients with normal pulmonary function undergoing general anesthesia with endotracheal intubation and transfusion of blood products should not be withheld from surgical patients as a means to prevent SSI [2, 7].

Our finding was also further investigated about the contributing factors of SSI after cesarean section. PROM > 12 h, duration of labor > 24 h, chorioamnionitis, anemia and having vertical skin incision all had increased risk of developing SSI after cesarean section.

Mothers who experienced PROM more than 12 h had increased risk of SSI than mothers who experienced PROM ≤12 h duration. This is possibly justified by that feto-placental membrane is one of the barrier essential for prevention or protection of ascending and iatrogenic infection of the membrane (chorioamnionitis). If this protective barrier is breached by any means, it will lose infection prevention. This can lead to ascending and iatrogenic (during per-vaginal examination) infections and will be bacteria reservoir (micro-organisms) and over growth. Unsterile membrane including the fluids which contain infection causing micro-organisms (such as bacteria) will have an access to other organs and tissues during cesarean section that can be potential source of infection after cesarean section. Supporting evidence was also reported from Egypt, India and Australia [39, 45, 46].

The other finding from this systematic review and meta-analysis also indicated mothers who had history of labor duration more than 24 h had increased risk of developing SSI than mothers whose labor duration was ≤24 h. Supportive finding was also reported from different country wide studies from India, China, Brazil and Nigeria [39, 41, 47, 48]. Hence, maternal early postpartum complication including infections (SSI) and exposure time where infection can be acquired increased as duration of labor increased. Beyond this, it is also the fact that prolonged labor along with increased number of vaginal examinations also increased the risk of SSI [49]. Labor pain is the severe form of pain causing maternal fatigue and dehydration as well as prolonged vascular diminishing to reproductive tract tissue by the presenting part which make favorable condition for microbes and infection even after the procedure.

Sectional having this evidences, this systematic review and meta-analysis also identified that chorioamnionitis another risk of SSI. Mothers who were diagnosed positive for chorioamnionitis had increased risk of SSI following cesarean section than mothers who don’t diagnosed for chorioamnionitis. Consistent finding was also reported from Canada, Australia and Estonia [38, 46, 50] . Chorioamnionitis is the inflammation of feto-placental membrane that can increase the chance of ascending or iatrogenic infection. This ascending infection can be complicated to sepsis for the both neonate and mother. This infection will affect or migrate to the sterile organs and tissues breached during cesarean section.

Moreover, anemia was also identified as medical factor which exacerbated SSI after cesarean section. Mothers who were diagnosed for anemia had increased risk of developing SSI after cesarean section than mothers who don’t diagnosed for anemia. This finding was consistent with a study conducted in Australia [46]. Anemia is one of the hematologic disorders that negatively affect mothers’ body infection protection mechanism or immune system. Iron is essential element for proper functioning of the host immune system. Low iron level during anemia alters the function of host immune system. In addition, low hemoglobin level causes lower oxygen saturation at peripheral tissue [51]. Delay in wound healing and low infection prevention finally leads to high risk of developing post procedure infection; SSI after cesarean section.

Once more, having history of vertical skin incision increases the risk of SSI. Mothers with history of vertical skin incision after cesarean section had increased risk of developing SSI than mothers with transverse skin incision. Comparable finding was also reported from Nepal [52]. The potential reason may be that, having vertical skin incision is associated with involving more areas, delayed wound healing, higher risk of wound dehiscence, that will put the mothers for risk of developing SSI following the procedure [53, 54].

In our study, a risk bias assessment showed that 7 (63.6%) studies had high quality scores and four (36.4%) had low quality scores. Representation and case-definition biases were the most commonly noted. To determine the influence of low methodological quality/high risk of bias on our estimates of pooled prevalence we estimated pooled prevalence without the low-quality studies. The confidence intervals of our estimates of pooled prevalence with and without these studies overlapped, indicating no significant difference between them. These results suggest that the majority of the primary study authors have met high quality standards. This lends credibility to our findings (Table 2).

### Limitation of the study

This systematic review and meta-analysis included only articles reporting in English language, which may restrict our findings. The majority of the articles use small sample size, might be affect the prevalence estimation. All included studies were cross sectional study design in which the result might potentially affected by confounding variables. In addition the meta-analysis didn’t include all regions and administrative city which only includes four regions and one administrative city of the country. Therefore further country based studies to assess other confounding factors related to health service factors, health policy factors and health care giver related factors for the prevalent SSI in Ethiopia is recommended.

## Conclusion

The prevalence of surgical site infection after cesarean section in Ethiopia was high compared with the sphere standards of CDC guidelines for SSI after caesarian section. Therefore, Ministry of Health with its stake holders should give special emphasis on community and institution based programs in manner to prevent prolonged labor, PROM, chorioamnionitis and anemia which will also have synergistic impact on caesarian section site infection. Moreover, there is also a call to health professionals not to use vertical incision as primary option of caesarian section to reduce the risk of surgical site infection among mothers.

## Supplementary information

**Additional file 1.** Preferred Reporting Items for Systematic Reviews and Meta-Analyses Protocols (PRISMA-P) checklist.

**Additional file 2.** Risk of Bias assessment Tool of Eligible Articles by using the Hoy 2012 tool.

## Data Availability

Data will be available from the corresponding author upon reasonable request.

## Abbreviations

AA: Addis Ababa
CDC: Center of disease control
HAI: Health care-associated infections
PPROM: Premature rapture of membrane
SNNPR: South Nation and Nationality peoples Region
SSI: Surgical site infection
